# Potentiation of anti-angiogenic eNOS-siRNA transfection by ultrasound-mediated microbubble destruction in *ex vivo* rat aortic rings

**DOI:** 10.1371/journal.pone.0308075

**Published:** 2024-08-01

**Authors:** Elisa Villa-Martínez, Amelia Rios, Roxana Gutiérrez-Vidal, Bruno Escalante

**Affiliations:** 1 Centro de Investigación y de Estudios Avanzados del Instituto Politécnico Nacional, Unidad Monterrey, Apodaca, Nuevo León, México; 2 Programa de Investigadoras e Investigadores por México, CONAHCyT/Centro de Investigación y de Estudios Avanzados del Instituto Politécnico Nacional, Unidad Monterrey, Apodaca, Nuevo León, México; Longgang Otorhinolaryngology Hospital & Shenzhen Key Laboratory of Otorhinolaryngology, Shenzhen Institute of Otorhinolaryngology, CHINA

## Abstract

Nitric oxide (NO) regulates vascular homeostasis and plays a key role in revascularization and angiogenesis. The endothelial nitric oxide synthase (eNOS) enzyme catalyzes NO production in endothelial cells. Overexpression of the eNOS gene has been implicated in pathologies with dysfunctional angiogenic processes, such as cancer. Therefore, modulating eNOS gene expression using small interfering RNAs (siRNAs) represents a viable strategy for antitumor therapy. siRNAs are highly specific to the target gene, thus reducing off-target effects. Given the widespread distribution of endothelium and the crucial physiological role of eNOS, localized delivery of nucleic acid to the affected area is essential. Therefore, the development of an efficient eNOS-siRNA delivery carrier capable of controlled release is imperative for targeting specific vascular regions, particularly those associated with tumor vascular growth. Thus, this study aims to utilize ultrasound-mediated microbubble destruction (UMMD) technology with cationic microbubbles loaded with eNOS-siRNA to enhance transfection efficiency and improve siRNA delivery, thereby preventing sprouting angiogenesis. The efficiency of eNOS-siRNA transfection facilitated by UMMD was assessed using bEnd.3 cells. Synthesis of nitric oxide and eNOS protein expression were also evaluated. The silencing of eNOS gene in a model of angiogenesis was assayed using the rat aortic ring assay. The results showed that from 6 to 24 h, the transfection of fluorescent siRNA with UMMD was twice as high as that of lipofection. Moreover, transfection of eNOS-siRNA with UMMD enhanced the knockdown level (65.40 ± 4.50%) compared to lipofectamine (40 ± 1.70%). Silencing of eNOS gene with UMMD required less amount of eNOS-siRNA (42 ng) to decrease the level of eNOS protein expression (52.30 ± 0.08%) to the same extent as 79 ng of eNOS-siRNA using lipofectamine (56.30 ± 0.10%). NO production assisted by UMMD was reduced by 81% compared to 67% reduction transfecting with lipofectamine. This diminished NO production led to higher attenuation of aortic ring outgrowth. Three-fold reduction compared to lipofectamine transfection. In conclusion, we propose the combination of eNOS-siRNA and UMMD as an efficient, safe, non-viral nucleic acid transfection strategy for inhibition of tumor progression.

## Introduction

The transport of water, nutrients, and proteins through vascular endothelium maintains tissue and fluid homeostasis. The endothelial cells (ECs) monolayer lining the inner surface of blood vessels are responsible for this function [1]. Nitric oxide (NO) is synthesized in the endothelium through the activity of endothelial NO synthase (eNOS) and has a central role in the regulation of vascular homeostasis. NO regulates changes in vascular tone, expression of adhesion molecules, platelet aggregation inhibition, endothelial cell growth, and redox processes [2]. NO is also an important mediator of angiogenesis [3]. Increased NO release from the endothelial cell monolayer is caused by a wide variety of vasoactive agonists as catecholamines, bradykinin, serotonin, arachidonic acid, or physical mechanisms as shear stress [4]. In fact, we and others have described the critical role of NO in the revascularization process after ischemia [5]. Guo *et al*. showed that constant production of endothelial NO by ECs promotes the development of collateral blood vessels during tissue ischemia [6]. eNOS knockout mice exhibit a limited angiogenic response to limb ischemia [7, 8]. Furthermore, NO has been established as a crucial signaling molecule and regulator of angiogenesis that acts as a downstream mediator of VEGF-triggered proliferation and migration of ECs [3]. Pharmacological agents that release NO, such as sodium nitroprusside and diazeniumdiolates (NONOates), also stimulate the proliferation and migration of ECs [9], thereby reinforcing the role of NO in the angiogenesis process. Thus, the local inhibition of eNOS to regulate microvascular permeability, vascular tone, and vasodilation may offer a promising approach as an anti-angiogenesis therapy [10]. Inhibition of NOS has also been proposed to inhibit tumor growth and vascularization [11, 12]. Indeed, increased expression of eNOS has been demonstrated in high-grade and metastatic castration-resistant prostate cancer [13]. eNOS and inducible *NOS* gene polymorphisms have also been associated with high susceptibility of aggressive prostate cancer [14, 15]. Thus, inhibition of NO offers a logical approach to reduce angiogenesis. Indeed, NOS inhibitors have been proposed as anti-angiogenic molecules. Down-regulation of eNOS expression by Cavtratin, a cell-permeable peptide that mimics Cav-1 functions, inhibited retinal neovascularization [16]. Also, inhibition of eNOS by L-NAME, a synthetic NOS inhibitor, led to the suppression of angiogenesis, cell proliferation and migration promoted by the NEDD4L overexpression in HUVECs [17]. However, these compounds are degraded in the systemic circulation requiring multiple doses to accumulate in the target site and increasing probability of side effects. Therefore, a more effective therapeutic approach is required to go beyond ameliorating symptoms by focusing on regulation of unfunctional genes. The use of nucleic acid-based therapies may potentially be the answer for the treatment of chronic diseases, more particularly silencing of eNOS gene may represent a good therapeutical approach to block NO-dependent angiogenesis. Activation or suppression of a specific gene represents an excellent opportunity to modulate the expression of the associated protein. In addition, use of genome editing tools like CRISPR/Cas9 system can result in permanent depletion of gene expression [18]. Through RNA interference (RNAi), small regulatory RNAs as siRNA or miRNA suppress RNA translation, also resulting in altered protein expression [18, 19]. However, to achieve effective and improved delivery of gene therapeutics, nucleic acids must successfully transfect the host cells and modify protein transduction. To reach these goals, nucleic acids must be shielded from degradation, maintain an appropriate concentration, and effectively internalize into the cytosol to reach the gene target [18, 20]. The crucial role of eNOS in the physiology of the vascular system and its ubiquitous distribution make the development of efficient siRNA delivery vehicles imperative, allowing controlled release in targeted areas. To date, viral vectors are the prevailing nucleic acid delivery vehicles. However, safety concerns about immunogenicity and toxicity of certain viruses have led to the development of non-viral delivery vehicles, including lipid-based delivery systems [18]. Several non-viral gene delivery vectors including Lipid (DOTAP), Cholesterol chloroformate, Dendrimer (PEI), and others, have been studied to increase nucleic acid delivery to various targets. PEI nanoparticles has been used to target tumor endothelial cells [21]. Efficient gene silencing in human coronary artery endothelial cells was also reported by Andersen *et al*. after multigene siRNA transfections, achieving high efficiency in protein knockdown [22]. In addition, cell/tissue-targeting functionalization of delivery vehicles has been approached to optimize endothelium delivery. Receptor-ligand binding to ECs surface proteins using monoclonal antibodies or peptides have been conjugated to delivery vehicles for targeting [18]. Thus, siRNA carriers to protect siRNA from degradation and increase localized delivery remain a challenge. Recently, milk-derived exosomes (mEXOs) were used as non-toxic siRNA carrier for Keap1 knockdown. This novel siRNA carrier accelerated wound healing as well as new vascularization in a model of diabetic mouse [23]. Although exosomes have been proposed as effective carriers in drug delivery, several issues as manufacturing due to the lack of a standard preparation methodology or low yield must be solved. More animal experimentation to detect probable adverse effects is also required before clinical translation [24].

In the recent years microbubbles (MBs) have emerged as efficient carriers for the delivery of drugs and genes. Lipid MBs are commonly used for contrast enhancement of ultrasound (US) in the evaluation of blood flow [25]. Positioning the US transducer in a specific tissue or organ allows visualization of the stimulated area [26]. The characterization of MBs interaction with US led to the finding that MBs could be loaded with therapeutic agents and then disrupted by low-frequency US to deliver the cargo [27]. This US-targeted MB destruction (UTMD) enhance the efficiency of gene transfection and drug delivery [28]. Several authors have reported UTMD to deliver antiangiogenic and cancer-related drugs. UTMD has also been used for gene transfection in animal models of cardiovascular diseases and cancer [28, 29]. Indeed, we demonstrated US-mediated MBs destruction (UMMD) as an efficient delivery system for the local release of a NO modulator associated with inhibition of vascular relaxation [30]. Furthermore, we demonstrated that the pharmacological effect of agonists or inhibitors of NO synthases, administered by UMMD, potentiated the biological effect of the drug [31, 32]. The use of MBs destruction by US has also been used to achieve an efficient transfection of siRNAs [33]. Suzuki *et al*. [34], demonstrated increased ICAM-1-siRNA transfection into injured mice arteries using the US-MB method. Wu *et al*. demonstrated improved myocardium blood perfusion by UTDM-mediated VEGF transfection. Moreover, gene transfection using this technology was associated with cytokine release and cardiac tissue regeneration [29]. Other authors demonstrated increased heart tissue adenoviral DNA transfection, suggesting that the combination of viral vectors with UTDM could be a therapeutic strategy to increase viral transfection efficiency in cardiovascular diseases [35]. Tumor growth inhibition has been recently demonstrated *in vivo* in a murine model of squamous cancer using a liposome-MB complex loaded with EGFR siRNA combined with US [36]. Thus, MBs as carriers for siRNA using ultrasound have proven effective in enhancing siRNA transfection and facilitating local release [37]. However, there are still some important issues which need to be addressed regarding acoustic parameters, particularly the potential safety, as well as a deep understanding of the complex interaction among US, MBs and ECs vasculature [38]. Hence, the aim of this study was to improve transfection and lower the dose required of eNOS-siRNA to prevent angiogenesis on rat aortic rings. eNOS-siRNA was loaded on MBs and released by US. We used bEnd.3 cells to assess the efficiency of eNOS-siRNA transfection with UMMD, the synthesis of NO using DAF2-DA, and eNOS protein expression by Western blot. The effect of silencing eNOS in angiogenesis was evaluated using the rat aortic ring assay.

## Materials and methods

### Reagents

DPPC (Dipalmitoyl-sn-glycero-3-phosphocholine), glycerol, 10% formalin solution, propylene glycol, bovine serum albumin (BSA), and L-NAME were purchased from Sigma-Aldrich (USA). DSPE-PEG2000 (1,2-distearoyl-sn-glycero-3-phosphoethanolamine-N- [methoxy(poly- ethylene glycol)-2000]), DSTAP (1,2-stearoyl-3-trimethylammonium-propane), and DC-Chol (3ß-[N-(N’,N’-dimethylaminoethane)-carbamoyl]cholesterol hydrochloride) were purchased from Avanti Polar Lipids (USA). Perfluoropropane gas (C_3_F_8_) was purchased from Coastal Specialty Gas (USA).

### Cell culture

Mouse brain microvascular endothelial cell line (bEnd.3 cells) was purchased from American Type Culture Collection (USA, cat. No CRL-2299). bEnd.3 cells were cultured in supplemented DMEM/F12 [10% (v/v) fetal bovine serum (FBS) and 1% penicillin/streptomycin] at 37°C in a humidified, 5% CO_2_ atmosphere. Experiments were performed with cells from passages 6 to 20.

### Preparation and characterization of microbubbles

Neutral and cationic lipid MBs were prepared using the mechanical agitation method as previously described [39]. Briefly, neutral lipids [DPPC: DSPE-PEG2000 (95:5, 5 mg/mL)] and cationic lipids mixture [DPPC: DSPE-PEG2000: DSTAP: DC-Chol (85:5:5:5, 5 mg/mL)] were dissolved in chloroform. DC-Chol was used to provide structural stability. Lipid films were obtained by evaporating chloroform under nitrogen. Then, the films were hydrated with buffer solution [phosphate-buffered saline (PBS, pH = 7.4), glycerol, and propylene glycol (80:10:10 v/v)] before all experiments. Then, the solution was transferred to an amber glass vial, degassed, and refilled with C_3_F_8_ gas. Finally, MBs were prepared via mechanical agitation (Ultramat S device, USA). MBs were washed twice for 20 min at 400g in a Sorvall Legend Microcentrifuge (Thermo Fisher Scientific, USA), and resuspended in buffer solution.

As previously described, the size distribution of MBs was evaluated by confocal laser microscopy (Leica TCS SP5, Germany) using a microfluidic chamber of polydimethylsiloxane (1×1×0.02 mm) [39]. The polydispersity index (PDI) was calculated as reported [40, 41]:

$$PDI=\frac{\sigma }{D_{m}}\times 100$$

where *σ* is the standard deviation and *D*_*m*_ is the average diameter. Nine images from three chambers were evaluated for three independent preparations. Confocal images were analyzed using the ImageJ software (National Institutes of Health, USA).

### Ultrasound exposure setup

In this study we utilized a mobile US device (Chattanooga Group, USA). The planar US transducer, active area of 5 cm^2^, was positioned at the base of a water bath at 37°C. Polystyrene, 24-well culture plates (well diameter = 15.6 mm) were placed 1 cm above the transducer surface. The cell monolayer was fully covered with 200 μL (2 mm in height) of culture media. Cells were exposed to sinusoidal US waves of 1 MHz, a duty cycle of 10%, and pulse repetition frequency (PRF) of 100 Hz for 1 min. The peak negative acoustic pressure (PNP) at different power intensities generated at the center of the transducer was independently measured with a calibrated HNR-0500 hydrophone (Onda, USA) in absence of the culture plate (S1 Fig). A synchronized digital oscilloscope was used to monitor the US signal (National Instruments, USA).

### Binding of siRNA to cationic MBs

A siRNA fluorescent universal negative control (6-FAM-siRNA, Sigma Aldrich, MO, USA) was used to demonstrate the binding of siRNA to MBs. Neutral and positively charged MBs (5 × 10^9^ MBs/mL) were previously stained with Vybrant DiI (V-22885, Molecular Probes, NY, USA) as described [30], then mixed with the negatively charged 6-FAM-siRNA for 15 min at room temperature (RT). The 6-FAM-siRNA-MBs complexes were diluted in buffer solution (1:10) and placed into microfluidic chambers, as above. Images were obtained using a Leica TCS SP5 laser confocal imaging system with excitation and emission wavelengths of 495/517 nm and 549/565 nm for 6-FAM-siRNA and Vybrant DiI, respectively.

### Determination of siRNA-loading capacity of MBs

The maximum amount of siRNA loaded on MBs was determined by incubating 5, 10, 20, or 40 μg of non-targeting siRNA (NC-siRNA; 5′-UGGUUUACAUGUCGACUAA-3′, Dharmacon, USA) with either neutral or cationic MBs at a concentration of 5 × 10^9^ MBs/mL for 15 min at RT. After centrifugation at 400 g for 5 min, non-bound siRNA in the subnatant was quantified by spectrophotometry (Nanodrop 2000, Thermo Scientific, USA). The amount of siRNA bound to MBs was calculated as the difference between the initial amount of siRNA and the amount quantified in the subnatant. The loading capacity per MB was calculated based on the amount of loaded siRNA for 2.50 × 10^8^ MBs. Loading efficiency (E) of siRNA-MBs was defined as:

$$E=\frac{Total\;amount\;of\;siRNA\;in\;the\;MBs}{Total\;quantity\;of\;siRNA\;added\;initially}\times 100$$

### Assessment of cell viability after UMMD

To find the appropriate UMMD parameters to avoid unwanted effects on cell viability, bEnd.3 cells were seeded on 24-well culture plates and divided into three experimental sets. In the first experimental set (Table 1 in S1 Table), cells were sorted in control group, without MBs or US exposure, and six other groups. Of these six groups, three were exposed to 1, 2 or 2.5 W/cm^2^ of intensity at 10% duty cycle, and the other three were exposed to the same intensities but at a 20% duty cycle. In the second experimental set (Table 2 in S1 Table), each intensity (0.50, 1 or 2 W/cm^2^) was evaluated with different ratios of MBs per cell (1:1, 4:1, 6:1, 8:1, or 12:1 MBs/cell). After adding the MBs, cells were immediately exposed to US at 10% of duty cycle.

In the third set (Table 3 in S1 Table), a ratio of 6:1 MBs/cell was exposed to 2 W/cm^2^ of intensity and evaluated at 0 and 24 h after treatment. Control groups, without MBs or US exposure or only exposed to US, were also evaluated at 0 and 24h after treatment. Cells were incubated at 37°C in a humidified 5% CO_2_ incubator. The frequency (1 MHz), exposure time (1 min), and PRF (100 Hz) remained unchanged for UMMD procedure. Following the treatment, cells were incubated in serum-free, fresh DMEM/F12 containing 0.10% ethidium homodimer (EthD-1) and 0.10% Hoechst 33342 (Molecular Probes, USA) for 30 min at 37°C. EthD-1 (ex/em 495/635 nm) is a high-affinity red fluorescent nucleic acid stain that selectively permeates through the compromised membranes of dead cells. The nucleic acid stain Hoechst 33342 (ex/em 350/461 nm) penetrates the cell and emits blue fluorescence when bound to nuclear DNA. Images were acquired using a Nikon Eclipse TE2000-U fluorescence inverted microscope (Nikon Instruments, USA). Ten fields per coverslip were captured at 10× magnification. Two coverslips were analyzed from each experimental group. Fluorescence images were processed using ImageJ software.

### Effect of UMMD on siRNA cellular uptake

To evaluate the transfection of siRNA using UMMD, fluorescent 6-FAM labeled siRNA universal negative control was used (Sigma-Aldrich). bEnd.3 cells were seeded on gelatin-coated coverslips placed into 24-well plates. After 48 h (60–80% cell confluency), culture media was replaced with 200 μL of fresh media containing 6-FAM-siRNA (44 ng) loaded-MBs and 2.5% FBS. Cells were then exposed to US for 60 s at 1 MHz, a PNP of 0.16 MPa (equivalent to a mechanical index of 0.16), a duty cycle of 10%, and a PRF of 100 Hz. After treatment, cells were incubated for 3, 6, 12, and 24 h at 37°C in a humidified 5% CO_2_ incubator. At the end of each incubation time, cells were fixed with 10% formalin (Sigma- Aldrich) for 20 min at RT. Fixed cells were labeled with the red fluorescent lipophilic membrane marker Vybrant DiI (5 μL/mL) and nuclei stained with Hoechst 33342 for 30 min at 37°C. Both markers were from Molecular Probes (USA). Then, coverslips were washed twice with PBS. Stained cells were mounted with Vectashield. Overlapping images were acquired at 400× magnification using a laser scanning confocal system (Leica TCS SP5). Transfection of 6-FAM-siRNA (44 ng) using Lipofectamine RNAiMAX (Thermo Fisher Scientific Inc., USA) following the manufacturer’s instructions was used as positive control of the experiment. Five images per coverslip were captured from two different coverslips from each experimental group, and the experiment was performed in triplicate. Fluorescence intensity from the green channel of confocal images was analyzed using ImageJ.

### UMMD-mediated eNOS-siRNA transfection

bEnd.3 cells at 60–80% cell confluency, were UMMD transfected with the sequence-specific eNOS-siRNA (UUGAGCUGGCUCAUCCACGdTdG, Sigma-Aldrich), or the NC-siRNA as negative control, in DMEM/F12 with 2.50% FBS. First, the effect of US intensity on the transfection efficiency of eNOS-siRNA was evaluated. Cells were incubated with eNOS-siRNA-loaded MBs, in a ratio of 6:1 MBs per cell, then exposed to four US intensities (0.25, 0.5, 1, and 2 W/cm^2^). The distance from the plate to the transducer, duty cycle, and exposure time remained constant. Subsequently, UMMD-mediated transfection of 42 ng of siRNA was compared with lipofectamine transfection using 42 and 79 ng of eNOS-siRNA. Cells were exposed to US for 1 min at 1 MHz, a PNP of 0.16 MPa (equivalent to a MI of 0.16), a duty cycle of 10%, and a PRF of 100 Hz. After treatments, cells were incubated for 24 h at 37°C in a humidified 5% CO_2_ incubator. Then reverse transcription quantitative PCR (RT-qPCR) and quantification of protein were assayed.

### Determination of siRNA gene-silencing efficiency

For RT-qPCR, cells were harvested 24h after transfection. Total cellular RNA was isolated from the cells using TRI Reagent (Sigma-Aldrich), quantified by spectrophotometry, and stored at -80° C until use. The experimental design is summarized in S2 Table. cDNA synthesis was carried out through reverse transcription using M-MLV reverse transcriptase and random primers (Invitrogen, USA). RT-qPCR was carried out with Fast Start Universal SYBR Green Master assay (Roche, USA) in a Step One Real Time PCR system (Applied Biosystems, USA). eNOS (NM_008713.4) and glucuronidase beta gene (Gusb, NM_001357025.1) primers sequence are as follows: 5’-GACCCTCACCGCTACAACAT (eNOS forward primer) and 5’CTGGCCTTCTGCTCATTTTC (eNOS reverse primer), 5’AGTTGTGGTATCCCAAGGGT (Gusb forward primer) and 5’GTCACCAGCCCGATGTCTTG (Gusb reverse primer). Each RNA sample was run in triplicate as an internal control. The relative levels of mRNA expression were quantified using the 2-ΔΔCT method [42]. Gusb was used as a reference gene. All primers were purchased from Sigma-Aldrich.

### eNOS protein expression

eNOS protein expression was quantified by Western blotting. The experimental design is summarized in S3 Table. Transfected and control cells were homogenized with lysis buffer (50 mM Tris-HCl pH = 7.4, 137 mM NaCl, 2 mM EDTA, 1% NP-40, 5% glycerol) supplemented with cOmplete, a protease inhibitor cocktail (Roche, USA). The supernatant was obtained by centrifugating the homogenate at 10,000 g at 4°C for 20 min. Total protein was measured by the BCA method (Thermo Scientific Inc, USA). Protein (40 μg) was resolved in an 8% SDS-PAGE. Mouse monoclonal anti-eNOS [M221] (1:500) and mouse monoclonal anti-actin [ACTN05 (C4)] (1:1000) antibodies, both from Abcam Inc. (USA), were detected by enhanced chemiluminescence (Amersham Biosciences, Germany) and analyzed with ImageJ.

### Nitric oxide detection

The experimental design to evaluate the inhibition of NO production induced by eNOS gene silencing using siRNA-loaded MBs and UMMD is summarized in S4 Table. NO imaging in transfected cells was performed with 4,5-diaminofluorescein diacetate (DAF2-DA, Enzo Life Sciences, USA). Cells were incubated with 5 μM of DAF2-DA in Ca^+2^ buffer for 30 min at 37°C, followed by the addition of 100 μM acetylcholine for 15 min. Cells used as negative control were preincubated with the eNOS inhibitor, NG-nitro-L-arginine methyl ester (L-NAME, 1 mM) for 1 h, then incubated with DAF2-DA. Cells were washed with PBS and fixed with 10% formalin for 20 min. Coverslips were washed twice with PBS, mounted with Vectashield, and visualized with a Leica laser confocal microscopy. Five images per coverslip were captured from two different coverslips from each experimental group, and the experiment was performed in triplicate. Fluorescence intensity from the green channel of confocal images was analyzed using ImageJ.

### Rat aortic ring assay

The rat aortic ring assay is a valuable, widely used model for studying *ex vivo* angiogenesis [43]. All the procedures conformed to the National Institutes of Health “Guide for the Care and Use of Laboratory Animals (1996)”. The protocol was approved by the Institutional Ethics Review Committee for Animal Experimentation of Cinvestav-IPN (approval no. 0170–15). Male Wistar rats (100–150 g of body weight) were fed a rodent standard diet and had water *ad libitum*. The rats were euthanized through cervical dislocation and all efforts were made to minimize the animal suffering. Afterwards the thoracic aorta was meticulously extracted and immediately immersed in serum-free DMEM/F12 within a 24-well plate. Aortas were carefully cleaned and cut into approximately 1 mm rings. Aortic rings were divided into three experimental groups: rings incubated without MBs or US (control), rings transfected with UMMD-mediated eNOS-siRNA, and rings transfected with eNOS-siRNA using lipofectamine. For UMMD-mediated transfection, eNOS-siRNA (131 ng)-loaded MBs (2.5x10^7^/mL) were added to the culture medium, then rings were exposed to US for 30 s at 1 MHz, PNP of 0.50 MPa (corresponding to a mechanical index of 0.50), a duty cycle of 10%, a pulse repetition frequency of 100 Hz. For lipofection, eNOS-siRNA (131 ng) and lipofectamine RNAiMAX were added to the culture medium following the manufacturer’s instructions. The aortic rings were then incubated overnight in serum-free medium at 37°C and 5% CO_2_. Thereafter, rings were carefully embedded in rat tail collagen type I (Corning, USA), let them stand for 15 min at RT and then incubated for 1 h at 37°C in a humidified 5% CO_2_ incubator.

Afterwards, the embedded rings were placed in DMEM/F12 supplemented with 2.50% FBS (v/v) and vascular endothelial growth factor (VEGF, 30 ng/mL, Sigma-Aldrich). A graphical representation of the experimental set up is included in S2 Fig. Quantitative evaluation of the angiogenic sprouting was determined by measuring the number of endothelial cell outgrowth from the primary tissue explant using MATLAB (MathWorks, USA). Between days five and six, aortic rings were directly imaged in the culture plate with a Nikon Eclipse inverted microscope. The initial process involved semi-automatically selecting the shape of the aortic ring by manually setting a threshold, which enabled the creation of a top hat filter to generate a suitable mask of this structure. The mask was then subtracted from image to keep only outgrowing cells. The next step involved quantifying the pixel density of the outgrowing cells while excluding the area covered by the aortic ring. This allowed for determination of the total number of cells outgrowing from the ring. In all experimental conditions, three fields per ring from three rings from each rat (n = 3 animals) were evaluated.

### Statistical analysis

Data are expressed as mean ± standard error of the mean or mean ± standard deviation. Differences between the two experimental groups were assessed using the Student’s t-test and the one-way analysis of variance for multiple comparisons. Data were evaluated using GraphPad (GraphPad Software, USA). A *P* value ≤ 0.05 was considered statistically significant.

## Results

### Characterization of microbubbles

**Fig 1:**
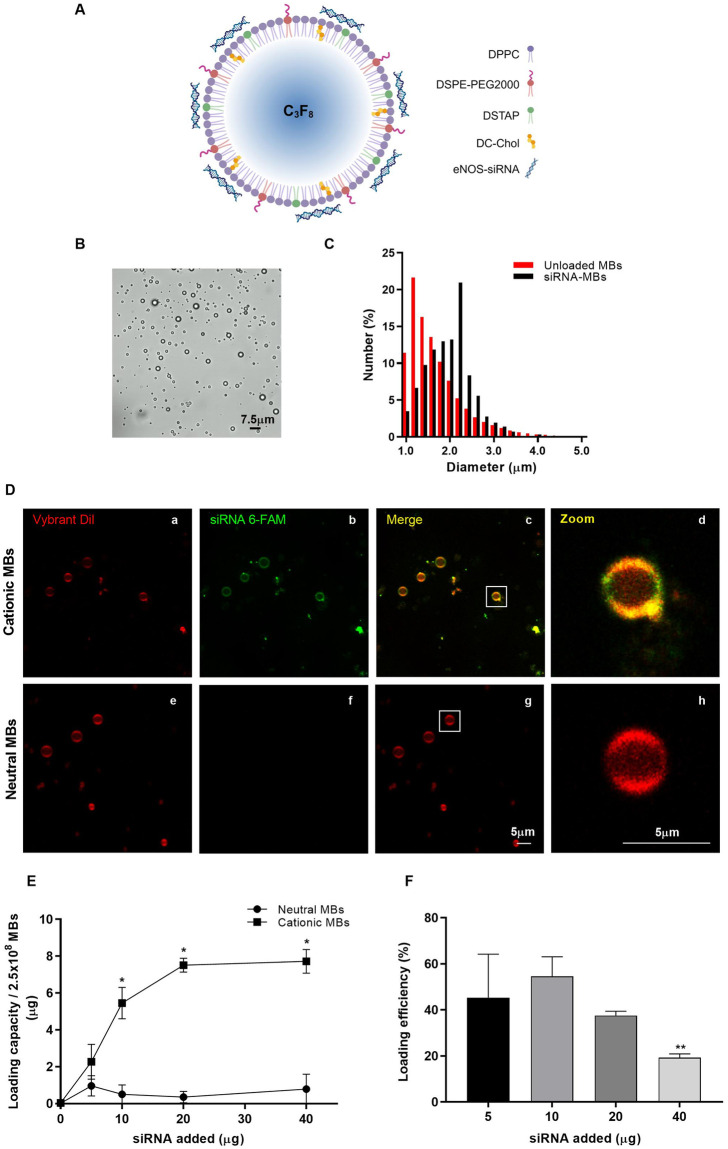
Characterization of microbubbles (MBs). (A) Schematic representation of cationic lipid MBs. The lipid composition of the MBs included DPPC, DSPE-PEG2000, DSTAP, and DC-Chol. DSPE-PEG2000 was utilized to improve the stability of MBs, while DSTAP and DC-Chol were incorporated to positively charge the surface and facilitate the binding of eNOS-siRNA through charge interactions. Diagrams were generated in BioRender (https://biorender.com; 2024). (B) Representative confocal image of siRNA-loaded MBs (siRNA-MBs). (C) The histogram represents the size distribution of cationic unloaded MBs and siRNA-loaded cationic MBs (siRNA-MBs). Three independent MBs preparations were analyzed. (D) Direct binding of siRNA to cationic MBs was visually confirmed by confocal microscopy. Cationic MBs loaded with the lipophilic membrane marker Vybrant Dil are observed as red fluorescence (a). 6-FAM-labeled siRNA is depicted as green fluorescence (b). Merge of red and green channels depicted as yellow fluorescence (c), showed colocalization of siRNA and the phospholipid monolayer of the cationic MBs. Neither binding of siRNA (f) or colocalization (g) was observed in neutral MBs. (E) Quantitative assessment of siRNA binding to cationic MBs. The siRNA loading capacity of cationic MBs was higher compared to neutral MBs as determined by the curve plateau. (F) Quantitative assessment of siRNA-loading efficiency. Data represent the mean ± standard deviation for three different experiments. (E) *P ≤ 0.0001 vs neutral MBs. (F) ** *P* ≤ 0.05 vs 10 μg.

Due to the anionic nature of siRNA, a MB formulation incorporating the positively charged lipids DC-Chol and DSTAP is proposed in this work, to facilitate the manufacturing of a siRNA-MBs through electrostatic interactions (Fig 1A).

The mechanical agitation method yielded a polydisperse suspension of 4.95 ± 0.90 × 10^9^ MBs/mL with an average diameter of 1.67 ± 0.62 μm (Fig 1B). The size distribution profile ranged from 1 to 4.8 μm with PDI of 34.90 ± 7.50%. The average diameter of MBs bearing siRNA was 1.96 ± 0.51 μm (Fig 1C). To demonstrate the incorporation of siRNA onto cationic MBs, the localization of siRNA was achieved by staining the lipid monolayer with the lipophilic membrane marker Vybrant Dil (Fig 1D). The stained membrane is depicted as red fluorescence (a) whereas the labeled-siRNA is visualized as green fluorescence (b). Merge of both channels was observed as yellowish fluorescence, indicating the colocalization of both membrane and siRNA (c). Confocal images visually confirmed binding of the fluorescent siRNA onto the cationic MBs (a-d), whereas neutral MBs were only stained with Vybrant Dil (g). Binding of siRNA or colocalization was not observed onto these MBs (e-h). To characterize the siRNA-loading capacity (Fig 1E) and loading efficiency (Fig 1F) of cationic MBs, four different concentrations of siRNA (5, 10, 20 and 40 μg) were evaluated. The saturation point was reached with 20 μg of siRNA that yielded a loading capacity of 7.50 μg in 2.50 × 10^8^ MBs, this is 30 ± 2 × 10^−9^ μg/MB (S5 Table). No significant differences were observed with higher concentrations of siRNA during following incubations. Based on these results, we selected the saturation concentration as the optimal amount of siRNA for use in subsequent experiments.

### Effect of UMMD on endothelial cell monolayer

**Fig 2:**
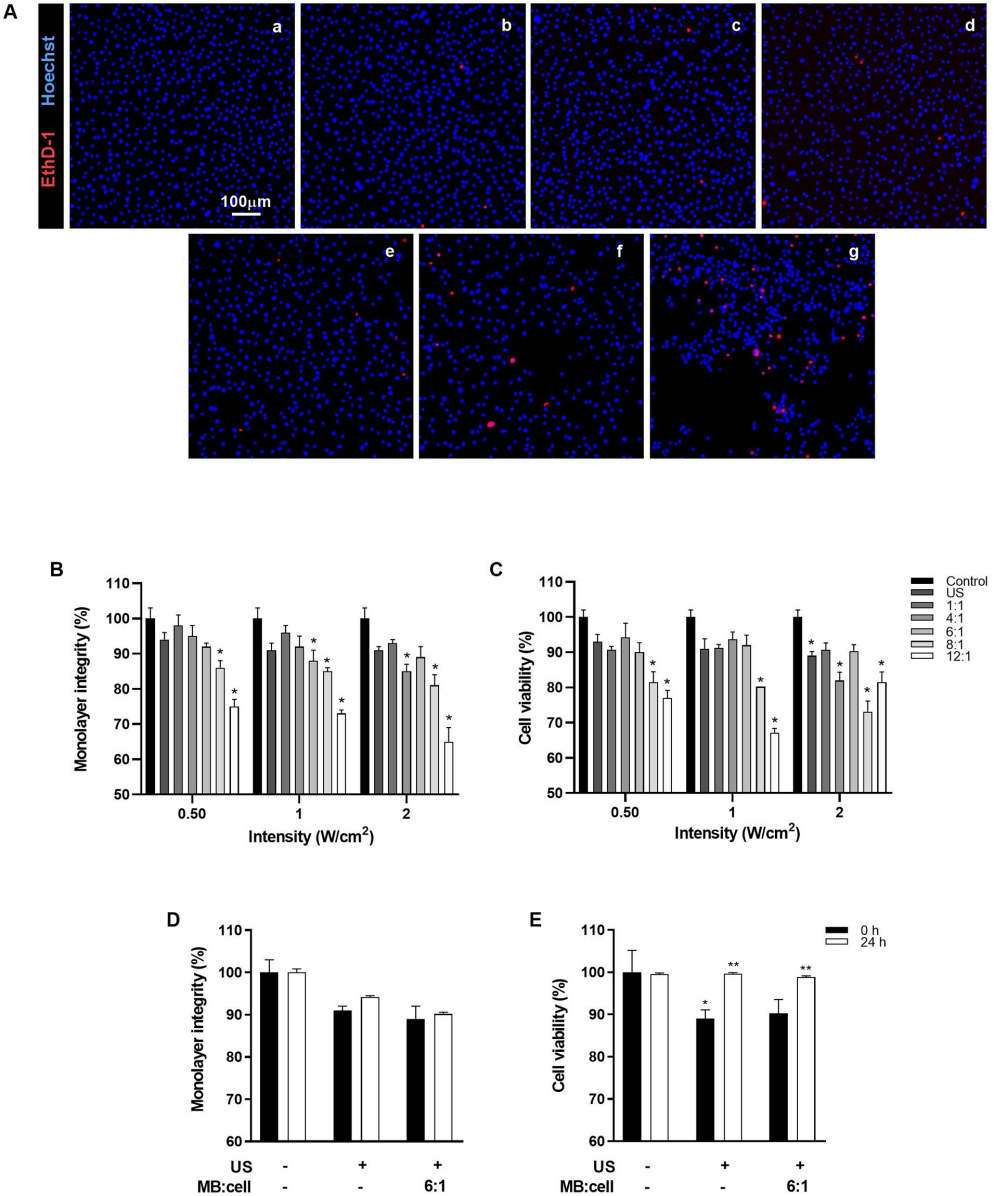
Monolayer integrity and cell viability following exposure to UMMD. (A) Representative fluorescence images of the endothelial monolayer exposed to US (0.50 W/cm^2^ of US intensity) using different MBs/cell ratios: (a) untreated cells, (b) cells exposed only to US, (c), (d), (e), (f), and (g) correspond to 1:1, 4:1, 6:1, 8:1, or 12:1 MBs/cell ratios, respectively. (B) Endothelial monolayer integrity and (C) cell viability were evaluated after UMMD treatment at 0.50, 1, and 2 W/cm^2^ US intensities using 1:1, 4:1, 6:1, 8:1, or 12:1 MBs/cell ratios. (D) Monolayer integrity and (E) cell viability at 0 and 24 h after UMMD treatment with a 6:1 MBs:cell ratio, 10% of duty cycle, and 2 W/cm^2^ of intensity. Data represent the mean of percentage ± standard error of the mean for three different experiments (n = 3). *P ≤ 0.05 vs control, **P ≤ 0.05 vs 0 h.

To identify the optimal US parameters required to achieve an efficient siRNA delivery while ensuring endothelial cell safety, we evaluated cell viability and monolayer integrity of bEnd.3 cells (Fig 2).

Fig 2A shows representative fluorescence microscopy images of cells after exposure to US using different ratios of MBs per cell (1:1, 4:1, 6:1, 8:1, or 12:1 MBs/cell) considering a monolayer of 250,000 cells. Control cells without exposure to MBs or US remained unaltered; the total population of nuclei is stained in blue (a). Cells exposed only to US suffered minimal damage (b). Alteration of the monolayer was increasing as the ratio MBs/cell increased: 1:1, 4:1, 6:1, 8:1, or 12:1, represented in (c), (d), (e), (f), and (g), respectively. This effect on the cells is depicted as red nuclei of dead cells and black empty spaces devoid of cells. The preliminary results evaluating duty cycle are described in S3 Fig. Fig 2B shows the effect of different MBs/cell ratios exposed to increasing US intensities (0.50, 1, and 2 W/cm^2^) with a PRF of 100 Hz, 10% duty cycle, and 1 min of time exposure. Monolayer integrity was assessed by calculating the percentage of adherent cells remaining after treatment, relative to the control group. The results revealed that monolayer integrity was not altered in control cells or cells exposed only to US. The ratio 1:1 MBs/cell did not affect the monolayer integrity at any of the three US intensities (0.50, 1, and 2 W/cm^2^). Increasing the ratio to 4:1 and 6:1 slightly decreased the monolayer integrity, however 89 ± 3% of the monolayer was preserved even at the highest intensity (2 W/cm^2^). Increasing MBs/cell ratio to 8:1 also slightly decreased the monolayer integrity with the highest effect at 2 W/cm^2^ (19 ± 3%). The major damage was observed with 12:1 MBs/cell ratio at 0.50, 1, and 2 W/cm^2^ US intensities (25 ± 2, 27 ± 1, and 35 ± 4%, respectively). Cell viability (Fig 2C), expressed as a percentage of the total population, was not affected using 1:1, 4:1, 6:1 MBs/cell ratios at 0.50 or 1 W/cm^2^ US intensities. However, with the 8:1 MBs/cell ratio, cell viability decreased by 19 ± 3% and 20 ± 0.01% at 0.50 and 1 W/cm^2^ US intensities, respectively. Exposure at 2 W/cm^2^ irregularly affected cell viability. Ratios of 1:1 and 6:1 MBs/cell had no effect whereas 4:1, 8:1, and 12:1 MBs/cell ratios decreased cell viability 18 ± 2, 27 ± 3, and 18 ± 3%, respectively. These results indicate a concentration-dependent effect of MBs on monolayer integrity and viability, allowing the selection of 6:1 MB/cell ratio for further experiments without negatively impacting the monolayer. Cell recovery after 24 h of UMMD treatment was next evaluated using a MBs:cell ratio of 6:1, duty cycle of 10%, and 2 W/cm^2^ of intensity. Treatment with US only or US+MBs reduced 10% the monolayer integrity, immediately after treatment. Cells treated with MBs maintained 90% integrity after 24 h (Fig 2D). Cell viability was also reduced 10% immediately after treatment with US or US+MBs, however after 24h, cell viability recovered up to 99% (Fig 2E).

### Efficiency of siRNA uptake after UMMD treatment

**Fig 3:**
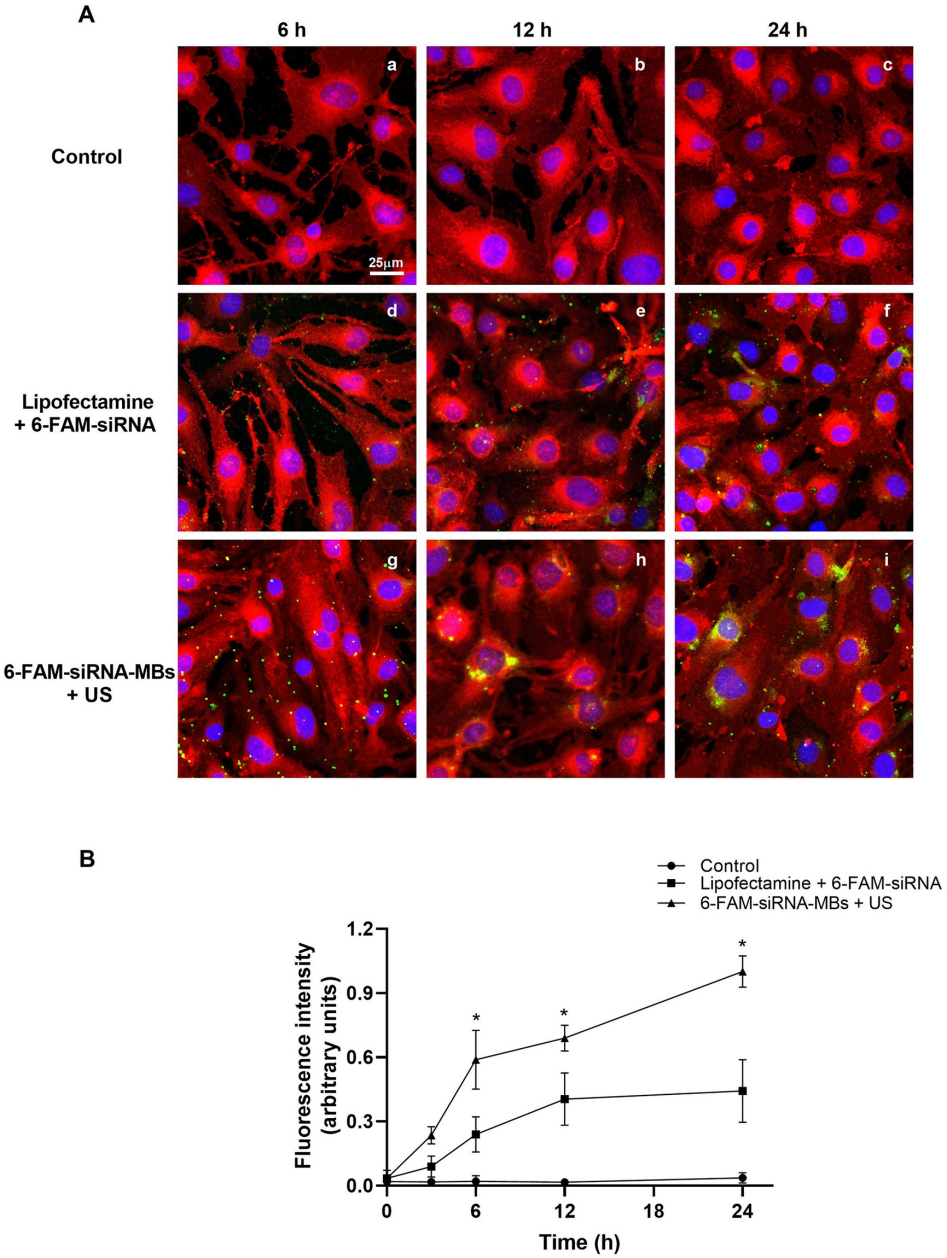
Delivery of 6-FAM-labeled siRNA assisted by ultrasound-mediated microbubble destruction (UMMD). (A) Confocal images revealed the intracellular uptake of 6-FAM siRNA (green) of UMMD-treated bEnd.3 cells as a function of time. Nuclei were stained with Hoechst 3342 (blue). Control without treatment after 6, 12 or 24 h (a, b, and c, respectively). Cells transfected using lipofectamine and 6-FAM siRNA at 6, 12 or 24 h after treatment (d, e, and f, respectively). Cells incubated with 6-FAM siRNA-MBs exposed to US at 6, 12 or 24 h after treatment (g, h, and i, respectively). (B) Fluorescence quantification of 6-FAM siRNA uptake. Data represent the mean ± standard error of the mean for three different experiments. *P ≤ 0.05 vs lipofectamine + NC-siRNA.

The delivery efficiency of MBs assisted by US on the transfection of siRNA was evaluated using a 6-FAM labeled siRNA at 6, 12, and 24 h (Fig 3).

Additionally, to confirm siRNA presence within cells, its localization was identified by staining cell membranes with Vybrant Dil (red). Representative composite images are shown in Fig 3A, indicating colocalization (yellow) of siRNA fluorescence (green) with cell membrane fluorescence (red). Confocal images demonstrated gradual increase of siRNA internalization after 6-FAM siRNA-MBs+ US treatment, with the higher amount of green fluorescence at 24 h after UMMD treatment. Quantitative analysis of green fluorescence showed that six hours post-transfection, the internalization of 6-FAM-siRNA + US (0.59 ± 0.07) was 2.50-fold higher than lipofection (0.23 ± 0.04), used as positive control of the experiment. Furthermore, this behavior continued until 24 h. Internalization using 6-FAM-siRNA (1 ± 0.04) + US was 2.30 -fold higher than lipofection (0.44 ± 0.08) (Fig 3B).

### *In vitro* evaluation of UMMD

**Fig 4:**
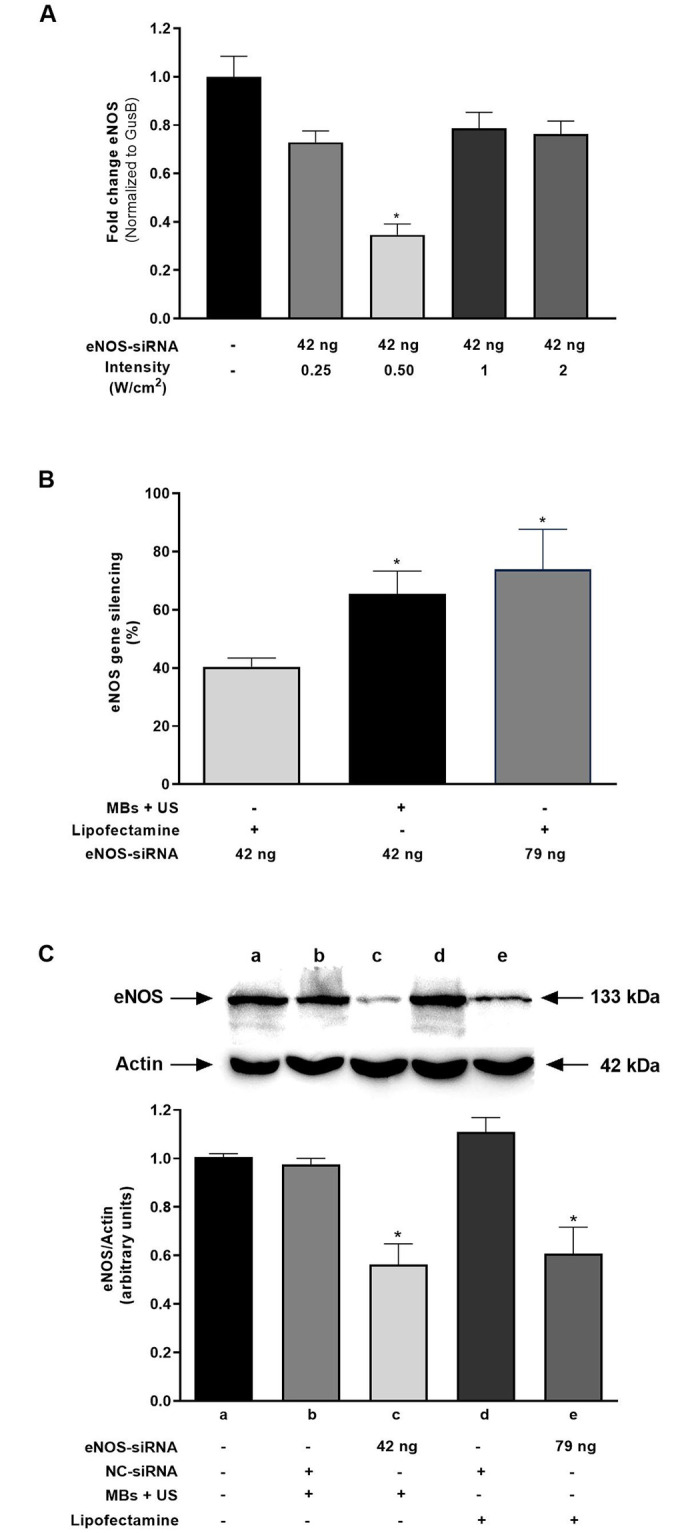
Comparison of eNOS-siRNA transfection delivered by UMMD and using lipofectamine on mRNA and protein expression. (A) Measurement of eNOS mRNA expression levels in UMMD-transfected cells using eNOS-siRNA-loaded MBs and 0.25, 0.50, 1, or 2 W/cm^2^ US intensities. (B) eNOS mRNA expression levels were evaluated by qPCR after 24 h of UMMD-transfection and lipofection. (C) eNOS protein expression levels were assessed by Western blotting 24 h post-transfection. Beta-actin was used as loading control. Data represent the mean ± standard error of the mean for three different experiments. (A and C) *P ≤ 0.05 vs control. (B) *P ≤ 0.05 vs lipofection 42 ng.

Next, we evaluated the effect of US intensity on the effectiveness of MBs in enhancing transfection of siRNA targeting eNOS. bEnd.3 cells were UMMD-transfected with the sequence-specific eNOS-siRNA (42 ng) using a MB:cell ratio of 6:1 and increasing US intensities. The distance from the culture plate to the transducer, duty cycle, and exposure time remained constant. As shown in Fig 4A, although eNOS expression level seemed to decrease at an intensity of 0.25 W/cm^2^, the difference was not statistically significative compared to control. However, at an intensity of 0.50 W/cm^2^ eNOS expression decreased 4.3-fold compared to control. In contrast, no differences were observed on eNOS expression levels with higher power intensity (1 or 2 W/cm^2^). Since the maximum effect was achieved at an intensity of 0.50 W/cm^2^, this value was used for subsequent experiments.

### UMMD-mediated eNOS-siRNA gene silencing efficiency

To evaluate the efficiency of eNOS silencing mediated by US and MBs, we compared mRNA and protein expression using two different concentrations (42 and 79 ng) of eNOS siRNA transfected with lipofectamine with transfection using eNOS siRNA-loaded MBs (42 ng) and US. Inhibition of eNOS mRNA relative expression expressed as percentage is displayed in Fig 4B. Transfection of 79 ng eNOS siRNA with Lipofectamine induced 74% inhibition of eNOS relative expression. However, it was determined in previous sections that the efficiency of siRNA binding to MBs was 0.03 pg/MB, corresponding to a calculated concentration of 42 ng based on the number of MBs. Consequently, when cultures are transfected with Lipofectamine at this concentration, only a 40 ± 1.70% relative inhibition is achieved. This percentage increased to 65.40 ± 4.50% using the same concentration of eNOS-siRNA released by MBs and US. Interestingly, the results demonstrate that using 79 ng resulted in a knockdown level of (73.90 ± 7.80%), reaching a level comparable to the effectiveness observed with UMMD.

### eNOS protein expression after UMMD-mediated gene silencing

The impact of eNOS gene silencing on protein expression was evaluated on UMMD- siRNA-transfected endothelial cells. Non-specific siRNA (NC-siRNA) a negative control, was used to assess any non-specific effects of siRNA internalization. As depicted in Fig 4C, no significant differences were observed between the NC-siRNA-treated groups and the control, indicating that the presence of an external nucleic acid or the transfection technique itself does not affect eNOS protein quantity. In contrast, transfection of 42 ng of eNOS-siRNA using UMMD reduced eNOS protein expression levels (52.30 ± 0.08%) to the same extent as 79 ng of eNOS-siRNA using lipofection (56.30 ± 0.10%). This is supported by the average relative density in both Lipofectamine and MBs-US transfected groups, being lower compared to the control group. Consistent with mRNA-level data, it is noteworthy that the silencing effect induced by both techniques is comparable. However, the MBs-US system requires a lower concentration of siRNA to achieve the same result.

### Effect of UMMD-mediated eNOS-siRNA on nitric oxide production

**Fig 5:**
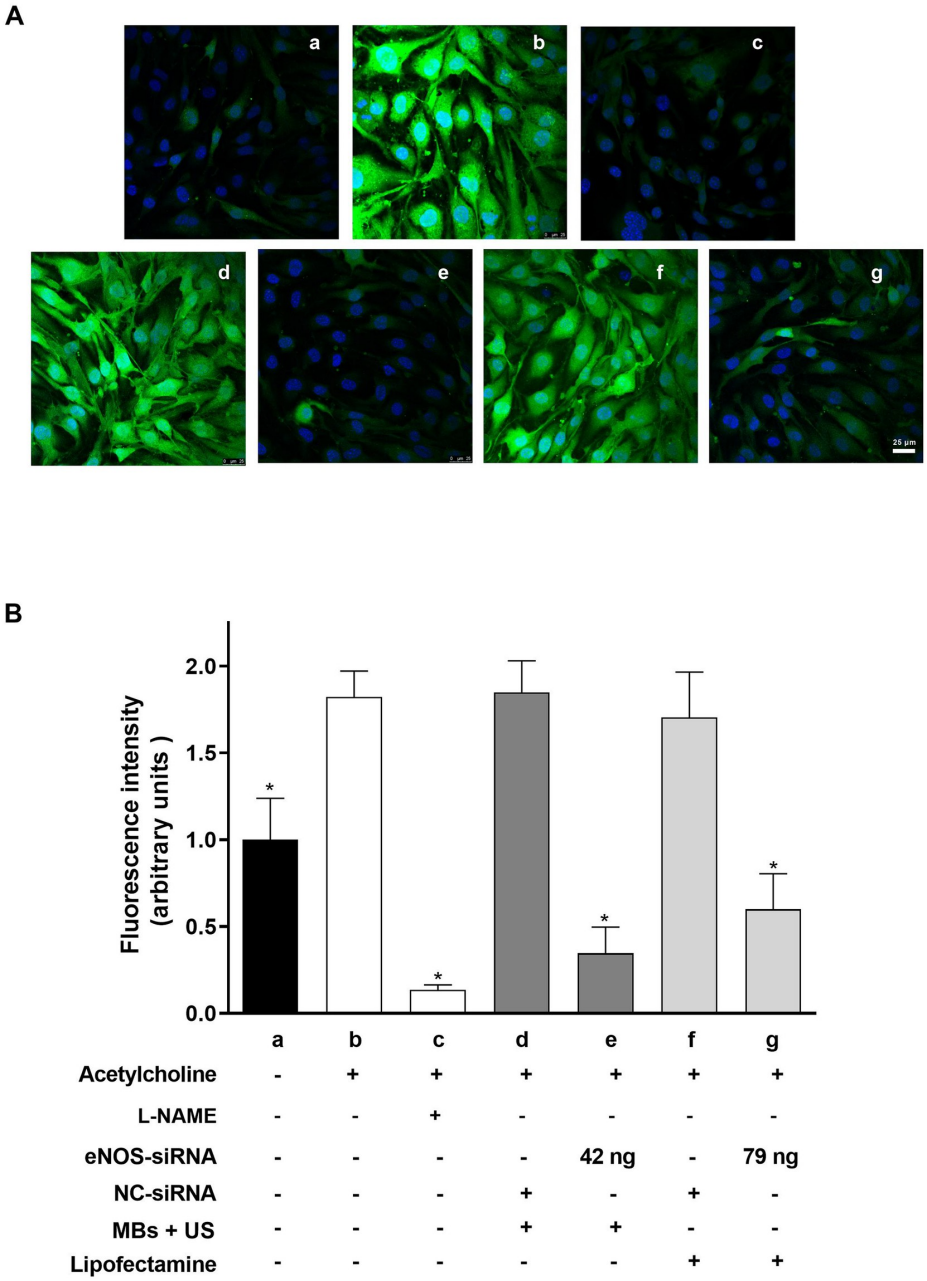
Transfection of eNOS siRNA by MBs and UMMD efficiently inhibits NO production in bEnd.3 cells. The upper panel (A) shows, representative confocal images depict endothelial NO release under different conditions: (a) Basal NO release; (b) Acetylcholine (Ach, 100 μM) stimulation; (c) Ach stimulation with NOS inhibitor L-NAME (1 mM); (e) Ach stimulation after transfection with eNOS-siRNA (42 ng)-MBs exposed to ultrasound (US); and (g) Ach stimulation after transfection with eNOS-siRNA (79 ng) using lipofectamine. NC-siRNA was used as a control for both UMMD and lipofectamine transfection (d and f, respectively). Quantification of fluorescence intensity (arbitrary units) of cells with or without Ach stimulation or treatment (control) is shown in panel (B). Each experimental condition was conducted in duplicate, and the entire experiment was replicated three times (n = 3). Data represent the mean ± standard error of the mean for three different experiments. *P ≤ 0.05 vs Ach.

To further corroborate the silencing of eNOS after UMMD treatment, the acetylcholine (Ach)-induced NO production was determined using the fluorescent NO indicator DAF2-DA. This highly sensitive and specific fluorescent indicator reacts with NO in the presence of oxygen to yield the highly fluorescent triazolofluorescein compound (Fig 5).

Representative images of NO production evidenced by green fluorescence in bEnd.3 cell line under different conditions are shown in Fig 5A. Confocal images showed basal NO production in control cells (a). The production of NO was then stimulated by addition of Ach (100 μM) (b). This response was inhibited by L-NAME (c). Transfection of eNOS-siRNA (42 ng) assisted by UMMD reduced the NO green fluorescence to levels approaching those observed with L-NAME (e). In contrast, lipofectamine-mediated transfection required 79 ng of eNOS-siRNA to achieve a similar level of knockout (g). The NC-siRNA control served as transfection negative control (d and f). Quantification of fluorescence is shown in Fig 5B. Ach and the NC-siRNA +Ach elicited the maximum NO production (1.82 ± 0.15 and 1.84 ± 0.18 arbitrary units, respectively). This effect was reduced in the cells transfected with eNOS-siRNA. However, transfection facilitated by UMMD (0.34 ± 0.15) decreased NO production by 81% compared to lipofection (0.60 ± 0.20) that reduced this production only 67%. In both cases, Ach was used as 100% of NO production. Thus, cells transfected with eNOS-siRNA-loaded MBs and US required 1.8 times less dose to induce a similar effect than lipofection. This result is consistent with our previous observations described above.

### Effect of UMMD-assisted eNOS-siRNA delivery on *ex vivo* rat angiogenesis model

**Fig 6:**
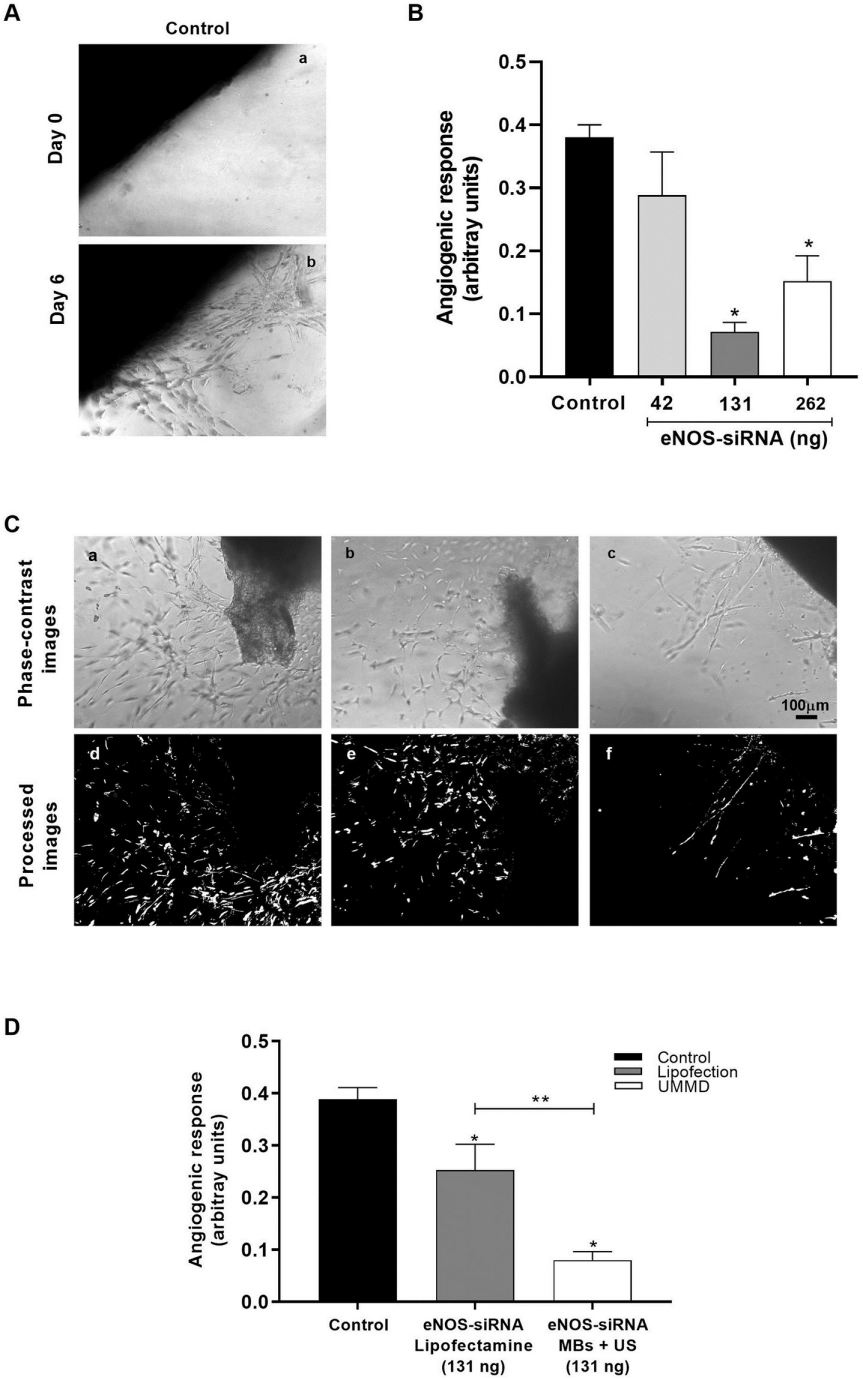
Effective inhibition of *ex vivo* angiogenesis mediated by eNOS-siRNA transfection using UMMD. The aortic ring assay was used as *ex vivo* model to evaluate angiogenesis in rats. (A) The image of an aortic ring at day 0 reveals the absence of microvessel sprouts. By day 6, EC proliferation becomes evident. (B) Characterization of the angiogenic response with respect to siRNA concentration. (C) Phase-contrast images of rat VEGF-treated aortic rings grown in type I collagen. (a) Control aortic rings, (b) aortic rings transfected using lipofectamine and 131 ng eNOS-siRNA, and (c) aortic rings treated with eNOS-siRNA loaded-MBs (131 ng) and exposed to US. (d, e, and f) corresponding processed images, respectively, showing in white the formation of new vessels. (D) Quantification of angiogenesis response using image analysis. Data represent the mean ± standard error of the mean for three different experiments (n = 3). *P ≤ 0.05 vs control, ** *P* ≤ 0.05 vs lipofection eNOS-siRNA.

After utilizing UMMD technology for delivery of eNOS-siRNA *in vitro*, we decided to assess the effect of eNOS siRNA transfection assisted by UMMD using an *ex vivo* approach. The aortic ring assay provides an *ex vivo* model to examine the complex process of angiogenesis. Culturing aortic rings from rats in a three-dimensional matrix, permits the evaluation of multiple factors involved in the formation of new blood vessels from pre-existing ones. Thus, the *ex vivo* angiogenesis was assessed using the rat aortic ring assay following the transfection of eNOS-siRNA facilitated by UMMD (Fig 6).

Phase-contrast images from rat aortic rings embedded in collagen and stimulated with VEGF at 0 and six days are shown in Fig 6A. At 0 days no vessel outgrowth was observed. Microvessel sprouting is clearly observed at day six. Next, we evaluated the angiogenic response using different doses of eNOS siRNA. Analysis of data demonstrated that transfection with 42 ng of eNOS-siRNA have no effect on the angiogenic response compared to control (0.28 ± 0.02 and 0.38 ± 0.06 arbitrary units, respectively). However, transfection with 131 (0.07 ± 0.01 arbitrary units) or 262 ng (0.15 ± 0.04 arbitrary units) of eNOS-siRNA resulted in inhibition of outgrowth compared to control (0.28 ± 0.02 arbitrary units). Interestingly, 131 ng of siRNA induced two-fold inhibition of sprouting compared to 262 ng (Fig 6B).

Transfection of eNOS-siRNA using lipofectamine or eNOS-siRNA loaded MBs and exposed to US, both reduced aortic ring outgrowth compared to control (Fig 6C).

Quantification of the angiogenic response by digital image processing using MATLAB (Fig 6Cd–6Cf) demonstrated that treatment with UMMD using eNOS-siRNA-loaded MBs reduced three-folds the angiogenic response (0.08 ± 0.01 arbitrary units) compared to transfection using Lipofectamine (0.25 ± 0.04 arbitrary units, Fig 6D).

## Discussion

In the present study, we demonstrated that UMMD using cationic MBs as eNOS-siRNA carriers improved siRNA transfection compared to the lipofection method.

**Fig 7:**
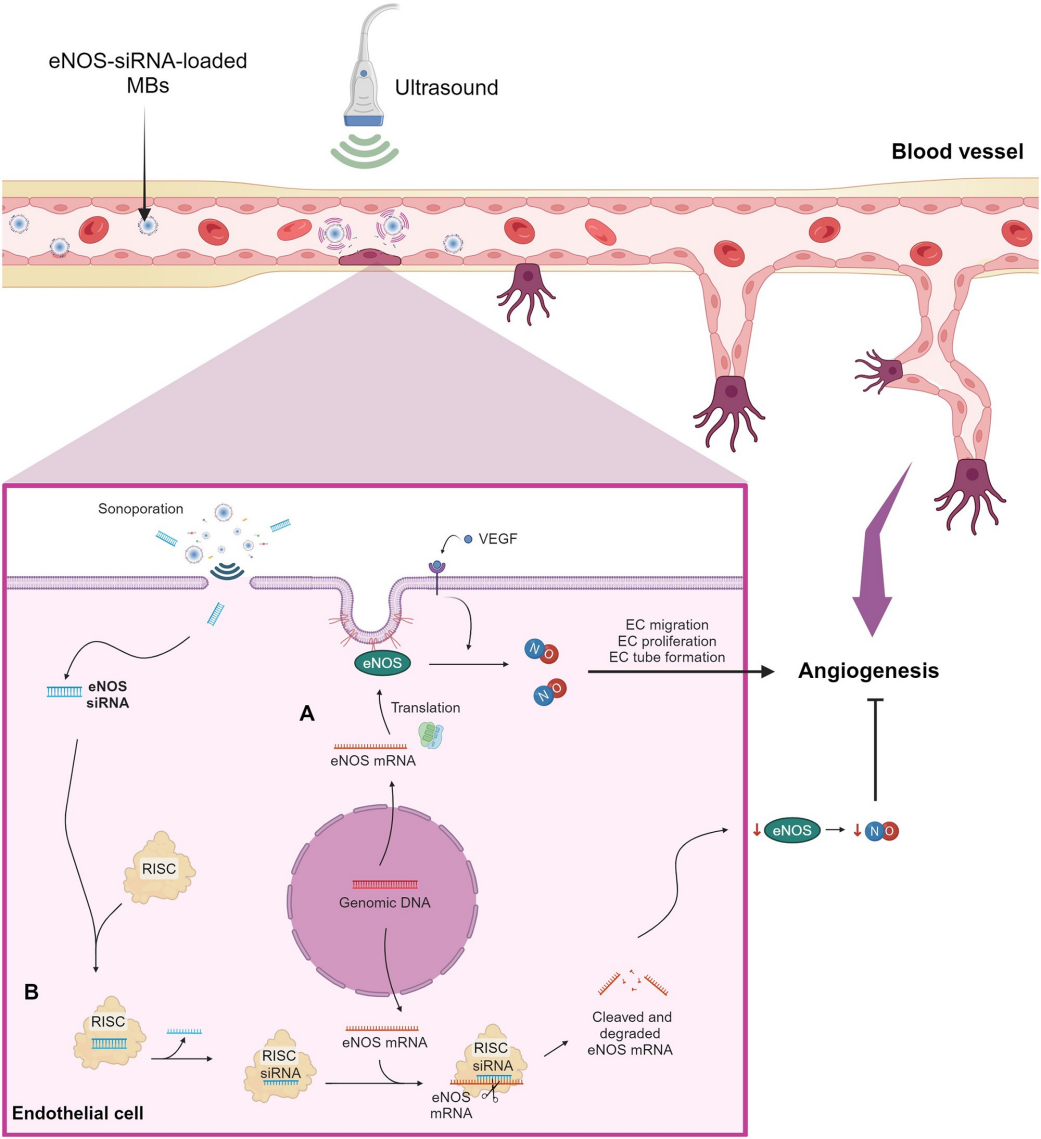
Schematic representation of eNOS-siRNA delivery by UMMD treatment to inhibit angiogenesis in blood vessels. (A) eNOS is constitutively expressed in endothelial cells. Stimuli such as Vascular Endothelial Growth Factor (VEGF) promote expression and activation of eNOS, inducing NO production. NO promotes endothelial cell migration, proliferation, and tube formation, which are crucial steps in the formation of new blood vessels (angiogenesis). (B) When MBs loaded with eNOS siRNA are directed towards the blood vessel and are stimulated with US, this US causes the rupture of MBs in a process called sonoporation, which facilitates the delivery of eNOS siRNA into the endothelial cells lining the blood vessel. Inside the endothelial cells, the eNOS-siRNA binds to the RNA-induced silencing (RISC) complex, leading to the cleavage and degradation of eNOS mRNA. This downregulates the expression of eNOS, resulting in decreased levels of NO and, consequently, inhibition of angiogenesis. Diagrams were generated in BioRender (https://biorender.com; 2024).

The efficacy of eNOS-siRNA delivery by UMMD is supported by our data, showing substantial reduction in mRNA and protein levels upon eNOS gene silencing. This effective decrease in gene expression corresponds to a notable decrease in NO production. Furthermore, an anti-angiogenic effect was achieved in rat aortic rings transfected with eNOS-siRNA. This effect was potentiated using UMMD technology compared to lipofectamine transfection. Thus, the combination of eNOS-siRNA and UMMD may represent an improved anti-angiogenic therapy for NO-dependent disorders associated with increased microvascular permeability and angiogenesis, particularly cancer angiogenesis [44]. Gene therapy is a medical approach to treat different diseases. Suppression of gene expression has been achieved with siRNAs in cultured cells and animals. HIV-1 infection of HeLa SX22-1 cells was inhibited by downregulation of CXCR4 expression [45]. Blazquez *et al*. demonstrated inhibition of the expression of target genes using a combination of U1 small nuclear RNA-snRNA-interference (U1i) and siRNA technology in tissue culture and mice [46]. However, the success of this therapy depends upon efficacy and safety of delivery vehicles. Our data showing binding of siRNA to cationic MBs are supported by previous reports that demonstrated the efficacy of cationic MBs as siRNA carriers [47]. However, some differences can be noticed. Binding of siRNA to MBs reached saturation with 20 μg, compared to the 40 μg of the short hairpin RNA plasmid reported by Zhang *et al*. Another difference was the maximal binding accounting for the number of MBs; our result showed 7.50 μg of siRNA per 2.50×10^8^ MBs compared to 17 μg of plasmid per 5×10^8^ MBs. These differences may be associated with composition and/or concentration of lipids utilized for the preparation of MBs. The relevance of increasing the payload is related with the number of MBs required to induce a biological effect. Lesser number of MBs has been associated with reduced damage to endothelial integrity [39]. Thus, to increase the siRNA payload capacity of MBs above 20 μg, several strategies may be explored. Variation in the lipid composition to enhance the loading capacity has been described by Zhang *et al*. [47], however the success of this strategy would depend on the high zeta potential that in turn may affect MBs stability. Another approach would be to increase MBs size thus increasing the surface available for binding of siRNA. A final proposal would be the use of liposomes conjugated to MBS. This delivery system combines the high loading capacity and targeting capacities of liposomes with the echogenic properties of MBs. We have previously reported a liposome-MB delivery system as hydrophilic agonist carrier to elicit and enhance vascular responses in rat aortic rings [31]. Similarly, a complex of cationic liposomes conjugated to MBs with a saturating payload of siRNA increased the delivery of EGFR-siRNA to carcinoma tumors [36]. Other cationic MBs with an improved plasmid-loading capacity have been reported [48]. However, characterization of those MBs cannot be compared with the data we reported here. The use of US and MBs for non-viral nucleic acids delivery has been reported by several groups [49, 50]. US-driven MBs response is crucial for drug/gene delivery processes [51]. Song *et al*. demonstrated enhanced gene transfection efficiency to spinal neurons using low-intensity focused US destruction of targeted cationic nanobubbles *in vitro* and *in vivo* [52]. Use of continuous US (mechanical index of 1.20) also enhanced eNOS gene transfection without compromising endothelial cell viability [53]. In addition, Suzuki *et al*., reported the effective, transfection of ICAM-1-siRNA in murine arteries, assisted by US-MBs [34]. US-mediated effective delivery of pDNA with poly(ethylene glycol)-SS-polyethylenimine-loaded MBs has also been reported as a potential gene therapy for ovarian cancer [54]. Moreover, Carson *et al*. achieved successful transfection of EGFR-siRNA utilizing MBs and US, leading to the inhibition of tumor growth in a mouse model of squamous cell carcinoma [55]. These studies highlight a promising strategy for cancer treatment that involves targeted gene therapy. Thus, in this work, we propose that siRNA delivery by MBs and US leads to enhanced transfection efficiency in bEnd.3 cells. This conclusion is supported by our data, that showed higher eNOS-siRNA gene silencing efficiency using UMMD (65.40 ± 4.50%) compared to lipofection (40 ± 1.70%). Moreover, protein and NO levels were reduced after eNOS gene knockdown assisted by UMMD. A previous study by Andersen *et al*. reported increased eNOS-siRNA transfection using distending pressure in human saphenous vein segments [56]. Despite the advantages of drug delivery with US, such as its non-invasiveness or low toxicity, this methodology still presents several limitations associated with low nucleic acids transfer efficiency or cell damage caused by US [57]. We have previously reported the optimal MB concentration and appropriate US parameters for the delivery of antennapedia-caveolin in both rat aortic rings [30], and cultured endothelial cells [32]. Here, we demonstrated that UMMD potentiated the silencing effect of eNOS-siRNA without detriment of cell function, reflected in a strong anti-angiogenic response in rat aortic rings. Angiogenesis is involved in pathologic conditions such as tumor growth or ischemic disease. An early report by Murohara *et al*. evidenced the relevance of eNOS in the neovascularization response to tissue ischemia [58]. Our results are consistent with a previous study that showed an impaired angiogenic response to tissue ischemia in mice with blocked eNOS by overexpression of caveolin-1 [59]. In addition, Cavtratin, an analog of caveolin-1, inhibited the proliferation of human umbilical vein endothelial cells (HUVECs), a key factor in vascular development and angiogenesis [60]. Thus, in this study we propose that delivering eNOS siRNA using MBs and US improves transfection efficiency in ECs thereby suppressing the angiogenesis process a crucial target in cancer therapy. This hypothesis is supported by our observation showing that transfection of 131 ng of eNOS-siRNA facilitated by UMMD, induced a two-fold inhibition of aortic rings sprouting compared to the transfection of a similar dose of eNOS-siRNA using lipofectamine (Fig 6). Thus, UMMD-mediated delivery of siRNA represents a useful therapeutic approach to avoid side effects, particularly when compared to systemically administered siRNAs. Fig 7 shows a graphical representation of the proposed mechanism involved in the inhibition of the angiogenic response through siRNA-eNOS-loaded MBs.

Although our data showed that UMMD improved siRNA transfection, our research has certain limitations. The payload capacity of MBs is still limited. The possibility of further increasing MB concentration could enhance the final dose of siRNA delivered to the target site. However, increased number of MBs would also lead to endothelial cell damage as observed in our results (Fig 2) and in a previous report [39]. Adjusting the US parameters can improve gene delivery efficiency. However, increasing parameters such as acoustic pressure or exposure have a detrimental effect on cell viability and gene delivery efficiency [61]. In addition, Sun *et al*. reported that delivery of AKT gene was improved increasing the cationic charge of MBs [48]. Thus, further investigation varying lipid composition and assessing the zeta potential is necessary. An additional limitation of this work is that this anti-angiogenic effect was not validated in pathological conditions in animal models. Nevertheless, our findings suggest a potential treatment approach for pathological conditions where eNOS-mediated angiogenesis is elevated, such as in tumor development. Indeed, regulating tumor vascular growth may be associated with metastasis as well limiting tumor size.

In conclusion, we demonstrated that eNOS-siRNA loaded into MBs and released by US is an efficient and safe non-viral nucleic acid transfection strategy with improved cellular specificity, thereby showing potential as anti-angiogenic therapeutic agent to improve tumor control. These findings contribute valuable insights into the potential of UMMD as a promising approach for targeted gene silencing, emphasizing the significance of the advance in the development of in advancing nucleic acid delivery strategies for cancer therapeutics.

## Supporting information

S1 Fig Characterization of the acoustic field as a function of power intensity. (PDF)

S2 Fig Schematic experimental design for assessment of the effect of UMMD-assisted eNOS-siRNA delivery on ex vivo rat angiogenesis. (A) Ultrasound exposure setup for aortic rings treatment. (B) After treatment, aortic rings are incubated at 37°C and 5% CO_2_ in serum-free medium for 24 h. Subsequently, aortic rings are gently embedded in a 3D collagen type I matrix, and angiogenesis was assessed by comparing the formation of sprouts from the treated and control aortic rings. Diagrams were generated in BioRender (https://biorender.com; 2024).
(JPEG)

S3 Fig Monolayer integrity and cell viability following exposure to UMMD. (A) Representative fluorescence images of the endothelial monolayer exposed to different US intensities: (a) untreated cells, (b), (c), and (d) are cells exposed to 1, 2, and 2.5 W/cm^2^ of intensity, respectively. (e) Zoom of the inset in (b). (B) Endothelial monolayer integrity and (C) cell viability were evaluated after exposure to 10% (black bars) or 20% (white bars) of duty cycle using 1, 2, and 2.5 W/cm^2^ of US intensity.
(JPG)

S1 Table Experimental design (set 1, 2 and 3). (PDF)

S2 Table Experimental design to evaluate eNOS gene silencing using UMMD. (PDF)

S3 Table Experimental design to evaluate the inhibition of eNOS protein expression induced by silencing eNOS gene using UMMD. (PDF)

S4 Table Experimental design to evaluate the inhibition of NO production eNOS-dependent induced by eNOS gene silencing using UMMD. (PDF)

S5 Table Loading capacity per MB. (PDF)

S1 Raw image Raw images of the anti-eNOS blot. (PDF)
