# Supplementary material for: Potentiation of anti-angiogenic eNOS-siRNA transfection by ultrasound-mediated microbubble destruction in ex vivo rat aortic rings
Source: PLoS One. 2024 Aug 1;19(8):e0308075. doi: 10.1371/journal.pone.0308075 (PMC11293687; doi:10.1371/journal.pone.0308075)
Supplement: S1 Fig — (PDF) [file pone.0308075.s001.pdf]

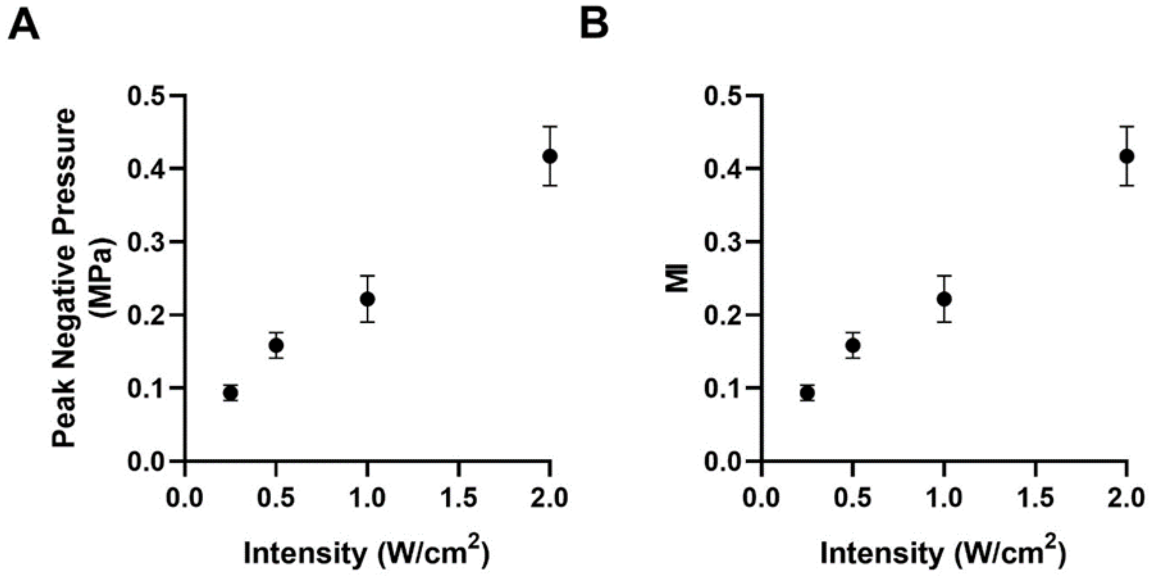

Fig S1. Characterization of the acoustic field as a function of power intensity. (A) Peak negative pressure as a function of power intensity. (B) Mechanical index (MI) as a function of power intensity. All measurements correspond to a frequency of 1 MHz and 10% duty cycle at a depth of 1 cm. Data represent the mean ± standard error of the mean for three different experiments (n = 3).
