# Supplementary figures and images for: Potentiation of anti-angiogenic eNOS-siRNA transfection by ultrasound-mediated microbubble destruction in ex vivo rat aortic rings
Source: PLoS One. 2024 Aug 1;19(8):e0308075. doi: 10.1371/journal.pone.0308075 (PMC11293687; doi:10.1371/journal.pone.0308075)

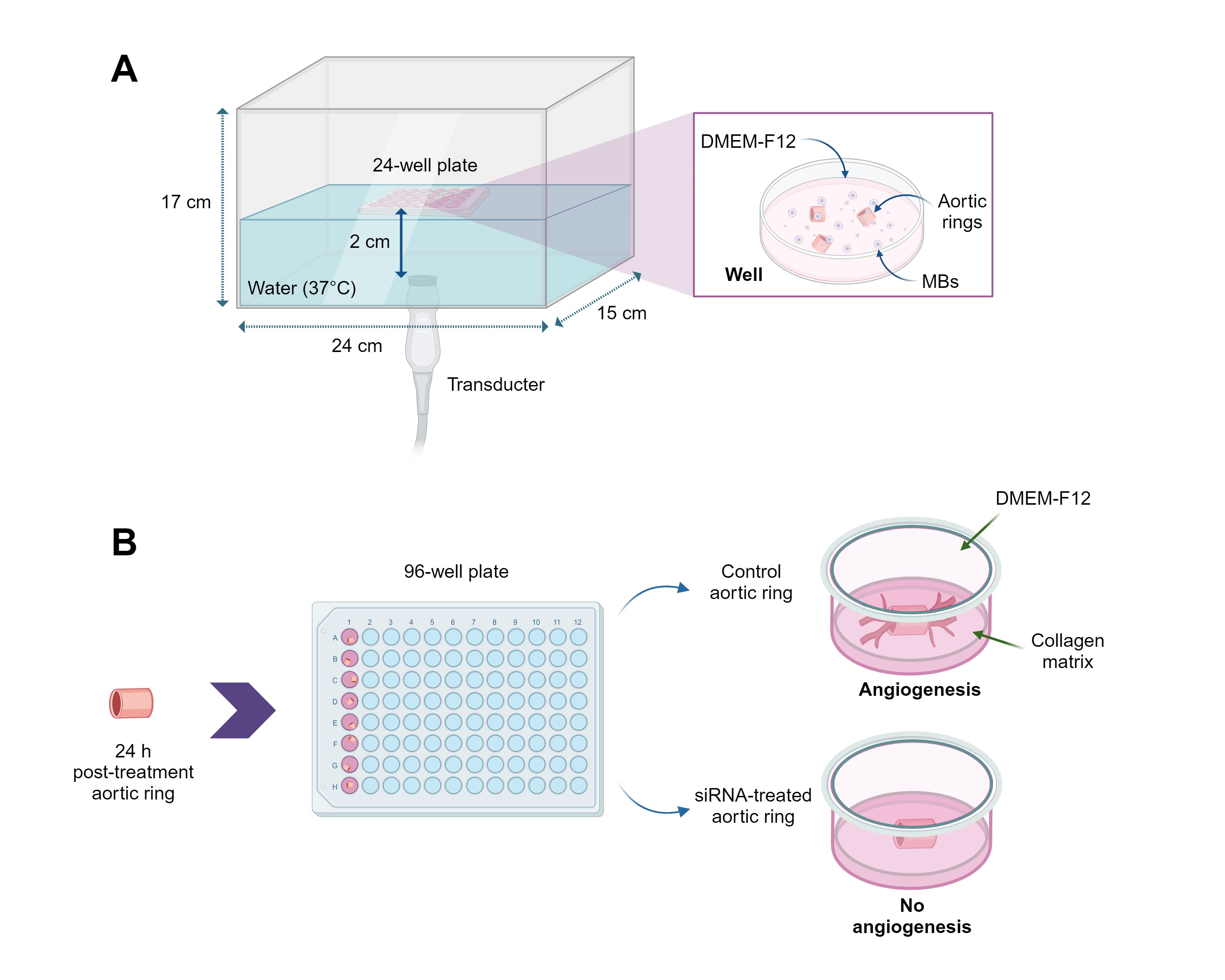

Supplement: S2 Fig — (A) Ultrasound exposure setup for aortic rings treatment. (B) After treatment, aortic rings are incubated at 37°C and 5% CO2 in serum-free medium for 24 h. Subsequently, aortic rings are gently embedded in a 3D collagen type I matrix, and angiogenesis was assessed by comparing the formation of sprouts from the treated and control aortic rings. Diagrams were generated in BioRender (https://biorender.com; 2024). (JPEG) [file pone.0308075.s002.jpeg]

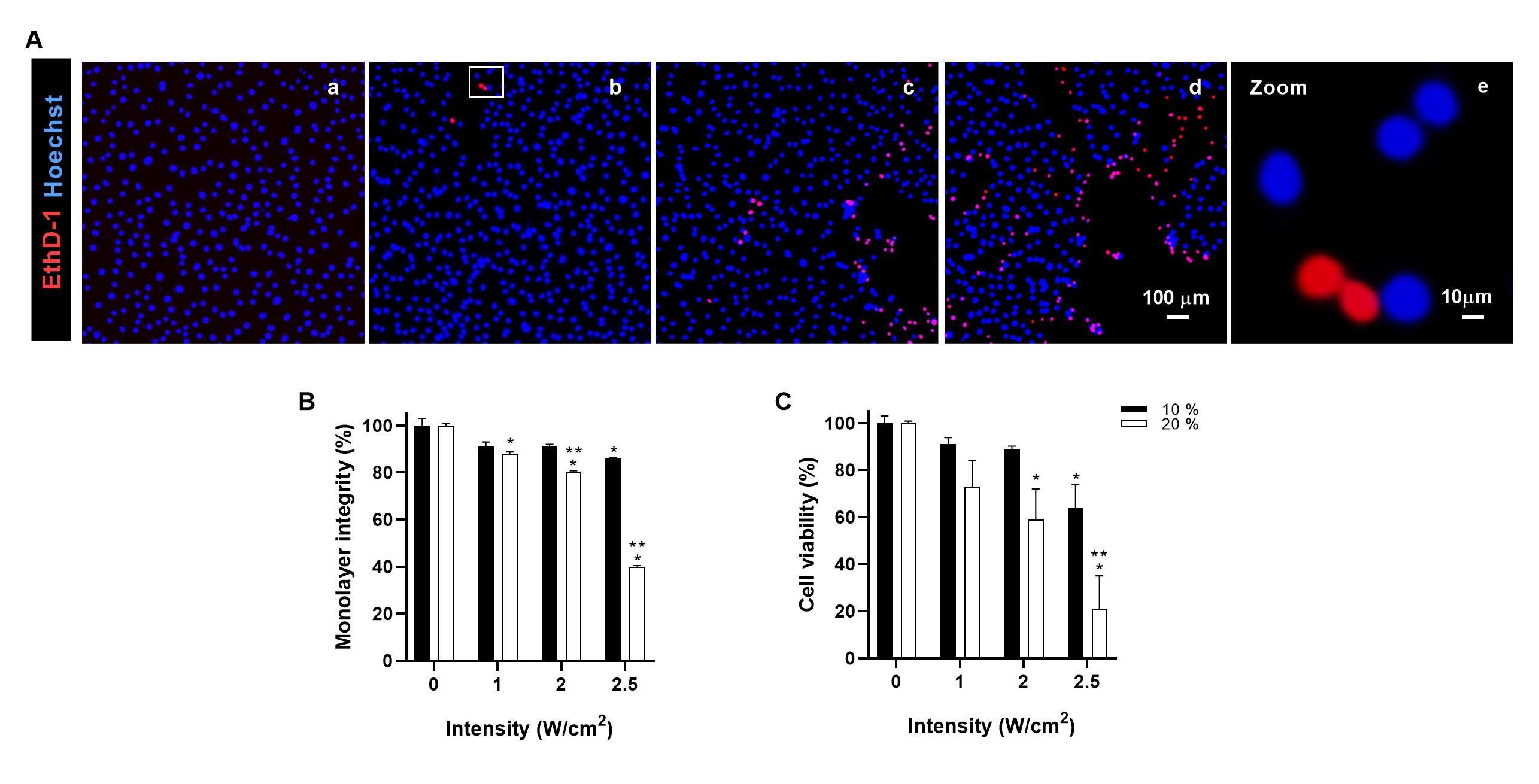

Supplement: S3 Fig — (A) Representative fluorescence images of the endothelial monolayer exposed to different US intensities: (a) untreated cells, (b), (c), and (d) are cells exposed to 1, 2, and 2.5 W/cm2 of intensity, respectively. (e) Zoom of the inset in (b). (B) Endothelial monolayer integrity and (C) cell viability were evaluated after exposure to 10% (black bars) or 20% (white bars) of duty cycle using 1, 2, and 2.5 W/cm2 of US intensity. (JPG) [file pone.0308075.s003.jpg]
