# Supplementary material for: Potentiation of anti-angiogenic eNOS-siRNA transfection by ultrasound-mediated microbubble destruction in ex vivo rat aortic rings
Source: PLoS One. 2024 Aug 1;19(8):e0308075. doi: 10.1371/journal.pone.0308075 (PMC11293687; doi:10.1371/journal.pone.0308075)
Supplement: S1 Table — (PDF) [file pone.0308075.s004.pdf]

Table 1. Experimental design (set 1) to evaluate cell viability and monolayer integrity after exposure to US.

US parameter		Group						
		1	2	3	4	5	6	7
DC (%)	10	-	+	+	+	-	-	-
	20	-	-	-	-	+	+	+
US Intensity (W/cm^2)	1	-	+	-	-	+	-	-
	2	-	-	+	-	-	+	-
	2.5	-	-	-	+	-	-	+

US = ultrasound; DC = duty cycle.

Table 2. Experimental design (set 2) to evaluate cell viability and monolayer integrity after UMMD treatment.

UMMD parameters		Group																		
		1	2	3	4	5	6	7	8	9	10	11	12	13	14	15	16	17	18	19
US Intensity (W/cm^2)	0.5	-	+	+	+	+	+	+	-	-	-	-	-	-	-	-	-	-	-	-
	1	-	-	-	-	-	-	-	+	+	+	+	+	+	-	-	-	-	-	-
	2	-	-	-	-	-	-	-	-	-	-	-	-	-	+	+	+	+	+	+
MBs:cell	1:1	-	-	+	-	-	-	-	-	+	-	-	-	-	-	+	-	-	-	-
	4:1	-	-	-	+	-	-	-	-	-	+	-	-	-	-	-	+	-	-	-
	6:1	-	-	-	-	+	-	-	-	-	-	+	-	-	-	-	-	+	-	-
	8:1	-	-	-	-	-	+	-	-	-	-	-	+	-	-	-	-	-	+	-
	12:1	-	-	-	-	-	-	+	-	-	-	-	-	+	-	-	-	-	-	+

MBs = microbubbles; US = ultrasound

Table 3. Experimental design (set 3) to evaluate cell viability and monolayer integrity after UMMD treatment.

UMMD parameters		Group					
		1	2	3	4	5	6
US Intensity (2 W/cm^2)		-	-	+	+	+	+
MBs/cell (6:1)		-	-	-	-	+	+
Time post-treatment (h)	0	+	-	+	-	+	-
	24	-	+	-	+	-	+

MBs = microbubbles; US = ultrasound
