# Supplementary material for: Potentiation of anti-angiogenic eNOS-siRNA transfection by ultrasound-mediated microbubble destruction in ex vivo rat aortic rings
Source: PLoS One. 2024 Aug 1;19(8):e0308075. doi: 10.1371/journal.pone.0308075 (PMC11293687; doi:10.1371/journal.pone.0308075)
Supplement: S2 Table — (PDF) [file pone.0308075.s005.pdf]

Table 4. Experimental design to evaluate the inhibition of eNOS mRNA expression induced by silencing eNOS gene using UMMD.

	Group 1	Group 2	Group 3
eNOS-siRNA	42 ng	42 ng	79 ng
MBs + US	+	-	-
Lipofectamine	-	+	+

eNOS = endothelial nitric oxide synthase; siRNA = small interfering RNA; mRNA = messenger RNA.
