# Supplementary material for: Potentiation of anti-angiogenic eNOS-siRNA transfection by ultrasound-mediated microbubble destruction in ex vivo rat aortic rings
Source: PLoS One. 2024 Aug 1;19(8):e0308075. doi: 10.1371/journal.pone.0308075 (PMC11293687; doi:10.1371/journal.pone.0308075)
Supplement: S3 Table — (PDF) [file pone.0308075.s006.pdf]

Table 5. Experimental design to evaluate the inhibition of eNOS protein expression induced by silencing eNOS gene using UMMD.

	Group 1	Group 2	Group 3	Group 4	Group 5
eNOS-siRNA	-	-	42 ng	-	79 ng
NC-siRNA	-	+	-	+	-
MBs + US	-	+	+	-	-
Lipofectamine	-	-	-	+	+

NC = Negative control; MBs = microbubbles; US = ultrasound
