# Supplementary material for: Potentiation of anti-angiogenic eNOS-siRNA transfection by ultrasound-mediated microbubble destruction in ex vivo rat aortic rings
Source: PLoS One. 2024 Aug 1;19(8):e0308075. doi: 10.1371/journal.pone.0308075 (PMC11293687; doi:10.1371/journal.pone.0308075)
Supplement: S5 Table — (PDF) [file pone.0308075.s008.pdf]

Table1. Loading capacity per MB

siRNA added (µg)	Loading capacity per MB (µg)
5	$9 \pm 4 \times 10^{-9}$
10	$22 \pm 3 \times 10^{-9}$
20	$30 \pm 2 \times 10^{-9}$
40	$31 \pm 3 \times 10^{-9}$

The loading capacity per MB based on initial amount of siRNA added (Mean ± standard deviation).
