# Supplementary material for: Potentiation of anti-angiogenic eNOS-siRNA transfection by ultrasound-mediated microbubble destruction in ex vivo rat aortic rings
Source: PLoS One. 2024 Aug 1;19(8):e0308075. doi: 10.1371/journal.pone.0308075 (PMC11293687; doi:10.1371/journal.pone.0308075)

Figure 4B
Anti eNOS 1:500
This image was obtained with a Micro Bioimaging System
DNR
MW 133 kD

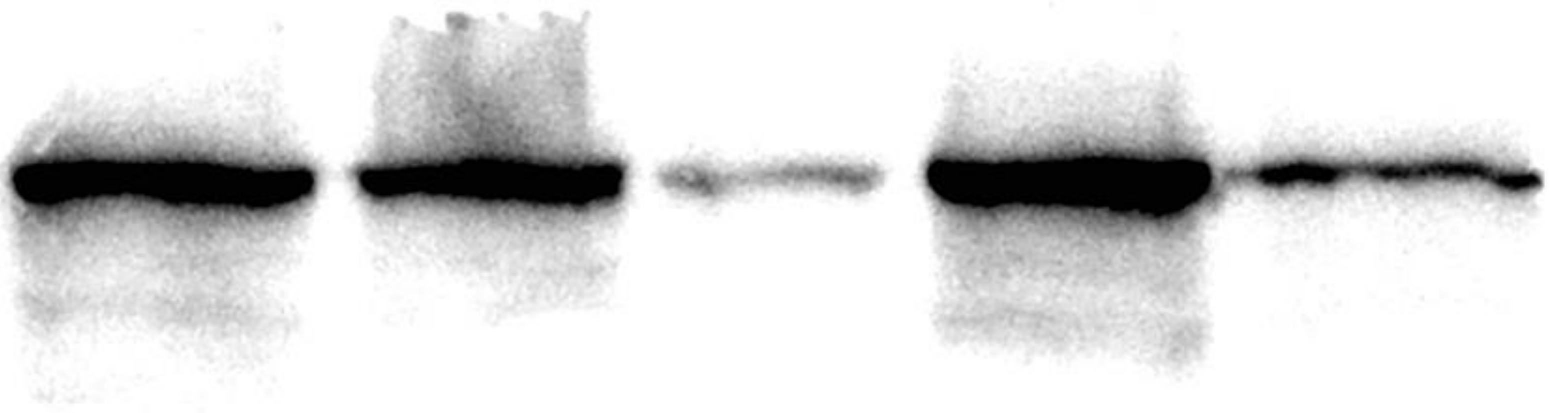

Figure 4B
Anti Actin 1:1000
This image was obtained with a Micro Bioimaging System
DNR
MW 42 kD

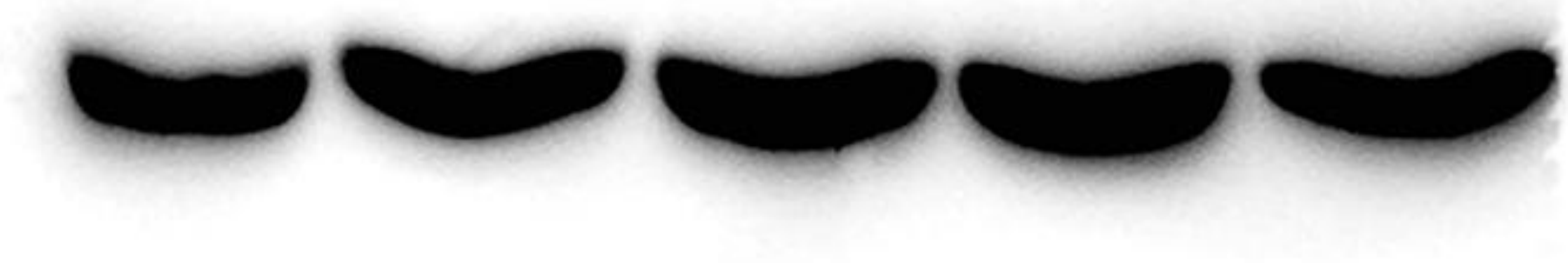

Supplement: S1 Raw image — (PDF) [file pone.0308075.s009.pdf]
